# Subcutaneous infliximab as a maintenance option in pediatric IBD: a real-world cohort including younger and lower-weight children

**DOI:** 10.1186/s40348-026-00251-2

**Published:** 2026-07-17

**Authors:** Neele Ridder, Johanna Overberg, Laura Kalveram, Samipa Pudasaini, Caroline Ruppel, Leonie Schumm, Stephan Henning, Philip Bufler

**Affiliations:** 1 Department for Pediatric Gastroenterology, Nephrology and Metabolic Diseases, Charité-Universitätsmedizin Berlin, Berlin, Germany; 2 Berlin Institute of Health at Charité – Universitätsmedizin Berlin, BIH Biomedical Innovation Academy, BIH Charité Clinician Scientist Program, Charitéplatz 1, 10117 Berlin, Germany; 3 German Center for Child and Adolescent Health (DZKJ), partner site Berlin, Berlin, Germany

**Keywords:** Infliximab, Subcutaneous, Pediatric ulcerative colitis, Pediatric Crohn’s disease

## Abstract

**Background:**

Subcutaneous infliximab (SC-IFX) offers an alternative to intravenous infliximab (IV-IFX) in adults with inflammatory bowel disease (IBD), but evidence in children is scarce. This study reports treatment persistence, tolerability, and pharmacokinetics in a real-world pediatric IBD cohort following transition from IV-IFX.

**Methods:**

We conducted a single center retrospective study including all pediatric IBD patients transitioned from IV-IFX to SC-IFX (120 mg every other week) at our institution between November 2023 and April 2025. Clinical disease activity scores, inflammatory markers, IFX serum concentrations, and anti-IFX antibodies (AIA) were assessed at baseline and during follow-up. The primary outcome was treatment persistence. Secondary outcomes included disease activity, pharmacokinetics, immunogenicity and tolerability.

**Results:**

Twenty patients (median age 14.5 years; range 5–17), including six children < 12 years and five weighing < 40 kg, were included. After a median observation period of 44 weeks (IQR 26–60), 16/20 patients (80%) remained on SC-IFX, with no significant difference between Crohn’s disease and ulcerative colitis. In a subgroup of four patients who received SC-IFX as a third dose following two intravenous induction doses, 3/4 (75%) maintained treatment over a median observation period of 60 weeks (range 16–64 weeks) and remained in clinical remission during follow-up. IFX serum concentrations increased after switching (median 11.8 µg/mL pre-switch vs. ≥24 µg/mL at follow-up), with most follow-up measurements reaching the assay ceiling. Concentrations were descriptively comparable between weight groups. All patients with detectable AIA prior to switching became antibody-negative during SC-IFX therapy, and no de novo antibodies were observed. Two patients discontinued therapy due to worsening of pre-existing paradoxical psoriasis. No other adverse events were documented.

**Conclusions:**

SC-IFX showed high persistence, stable inflammatory markers and good tolerability in this pediatric cohort, including younger and lower-weight children. SC-IFX appears to be a feasible maintenance option in selected pediatric patients, including early use after induction. Prospective studies are warranted to define pediatric-specific pharmacokinetic targets and individualized dosing strategies.

**Supplementary Information:**

The online version contains supplementary material available at 10.1186/s40348-026-00251-2.

## Background

 The global incidence and prevalence of pediatric inflammatory bowel disease (IBD) is steadily increasing [1]. Effective and practical treatment strategies are required to ensure optimal disease control and improve patient quality of life. Infliximab (IFX), a chimeric anti–tumor necrosis factor (TNF) antibody, has been established as a standard therapy with good efficacy in pediatric IBD [2, 3]. According to international guidelines, IFX is administered intravenously (IV-IFX) in children and adolescents with moderate to severe Crohn’s disease (CD) and ulcerative colitis (UC) [4, 5].

For adult IBD patients (aged ≥ 18 years), subcutaneous IFX (CT-P13, RemsimaⓇ, Celltrion Healthcare, Incheon, South Korea) (SC-IFX), administered at a dose of 120 mg every other week, has recently demonstrated good efficacy and safety as maintenance therapy with a treatment persistence of 92–100% after 12 months [6, 7]. Thus, SC-IFX expands the therapeutic options tailored to individual patient preferences and circumstances. Despite its potential advantages—including fewer hospital visits, reduced school absenteeism, avoidance of intravenous cannulation, and decreased burden on healthcare systems—SC-IFX is not yet licensed for pediatric use.

To date, evidence regarding the efficacy and safety of SC-IFX in pediatric IBD remains limited. Existing data are mainly derived from a small number of observational studies, largely focusing on adolescents (≥ 15 years) or children with higher body weight (≥ 40 kg) [8–10]. More recently, Eldredge et al. reported the first prospective pediatric study on SC-IFX over a relatively short observation period of 12 weeks [11]. While recent studies have expanded the available evidence [10], detailed data on long-term treatment outcomes, pharmacokinetics, and immunogenicity—particularly in younger and lower-weight pediatric patients—remain limited.

To further complement the existing literature, we conducted a retrospective study evaluating the efficacy, tolerability, and pharmacokinetic profile of SC-IFX in a broader pediatric IBD population following transition from IV-IFX.

## Methods

### Patients and study design

We conducted a single center retrospective cohort study between November 2023 and April 2025 at Charité-Universitätsmedizin Berlin. All pediatric IBD patients (< 18 years) receiving SC-IFX during this period were included in the study. The study was approved by the local ethics committee (EA2/094/25).

All patients who had received at least two doses of IV-IFX were eligible to be switched to SC-IFX, with adolescent patients and patients in clinical remission (pediatric ulcerative colitis activity index (PUCAI) < 10 or short pediatric Crohn’s disease activity index (shPCDAI) ≤ 15) being prioritized. Patients eligible for a switch were informed about the off-label use of SC-IFX in this age group and elective switching was at the joint decision of patients, parents and the treating clinician. Patients with known anti-IFX antibodies (AIA) were also included in our study.

All patients, regardless of weight, previous dosing regimen and interval of IV-IFX, were switched to 120 mg SC-IFX (CT-P13, Remsima) every other week according to the official product information for IFX.

The first SC-IFX dose was administered 4 weeks after the last IV-IFX infusion (baseline visit V0), when IV-IFX trough concentrations were obtained. Subsequent SC-IFX doses were self-administered, and blood sampling was performed at follow-up visits without timing adjustment to the injection schedule; these values were therefore reported as random serum concentrations.

Data were collected at the baseline visit (V0), the first follow-up visit (V1, median 4 weeks post-switch, IQR 4–4), the second follow-up visit (V2, median 16 weeks post-switch, IQR 14–20), and the last visit (VL, median 44 weeks post-switch, IQR 26–60). Given the modest peak-trough fluctuation of SC-IFX at steady state [12, 13], V2 random serum concentrations are considered to approximate steady-state exposure.

### Data collection

We collected baseline clinical data (age, height, weight, sex), IBD characteristics (IBD subtype, Paris classification), prior treatment and current IV-IFX treatment regimen (IV-IFX dose, interval and concomitant medication, time on IV-IFX). To assess clinical disease activity, the PUCAI was calculated for UC and the shPCDAI for CD. Furthermore, we assessed laboratory results (albumin, C-reactive protein (CRP), fecal calprotectin levels), IFX concentrations and AIA at baseline (V0) and follow-up (V1, V2). Clinical remission was defined by PUCAI < 10 and shPCDAI ≤ 15.

IFX serum concentrations and AIA were measured in serum samples using a chemiluminescence immunoassay (CLIA) with the i-Tracker IFX assay and the i-Tracker anti-IFX assay (Biosynex–Theradiag, Croissy-Beaubourg, France). IFX concentrations were quantifiable within a range of 0.3–24 µg/mL; values below the lower limit of quantification (LLOQ, < 0.3 µg/mL) were plotted at zero for visualization only. AIA were classified as positive (≥ 10 ng/mL) or negative.

### Outcome measures

The primary outcome was treatment persistence at the last follow-up visit (VL). Secondary outcome variables were IFX concentrations and antibodies, disease activity and inflammatory markers before and after switching to SC-IFX. Adverse events were captured by reviewing the electronic medical record, including routine follow-up consultations.

### Statistical analysis

Effect size calculation was based upon the one-sided 95% confidence interval (CI) for an assumed treatment persistence rate at VL of 92%: With a sample size of *n* = 20, this confidence interval would extend 10% in the lower direction. Effect size calculation was performed using the commercial software nQuery + nTerim, version 4.0 (Statistical Solutions Ltd, Cork, Ireland).

Descriptive statistics were used for demographics and baseline characteristics, summarizing normally distributed data as mean and standard deviation (SD) and non-normally distributed data as median and interquartile range (IQR). Categorical variables were summarized as absolute and relative frequencies (%). For the primary endpoint *treatment persistence rate at VL*, the one-sided 95% CI was calculated. Non-normally distributed data were analyzed using the Wilcoxon signed-rank test for paired samples and the Mann-Whitney U test for independent samples. Categorical variables were compared using Fisher’s exact test when expected cell counts were less than five. Treatment persistence was estimated using the Kaplan-Meier method. Serum IFX concentrations were analyzed descriptively only — both for the pre- versus post-switch comparison (V0 vs. V2) and for the comparison between weight groups (< 40 kg vs. ≥40 kg) — because the small sample size and the right-censoring of values at the upper limit of quantification (ULOQ) of the assay (≥ 24 µg/mL) preclude valid inferential testing. IFX concentrations are therefore reported as medians with ranges and as the proportion of values at or above the assay ceiling; where more than half of the values reached the ceiling, the median is reported as a lower bound (≥ 24 µg/mL). Body surface area (BSA) was calculated using the Haycock formula [14]. For the exploratory analysis of pre-switch IV-IFX dose intensity per BSA (DI/BSA) versus V2 serum IFX concentrations, patients were dichotomized by whether V2 concentrations were at or above the ULOQ (≥ 24 µg/mL) or below, and groups were compared using the Mann-Whitney U test. Statistical analyses were conducted using SPSS software, version 30.0.0.0 (IBM Corp., Armonk, NY, USA).

## Results

### Patient characteristics

Overall, 20 patients (nine with UC and 11 with CD) agreed to switch to SC-IFX. There were no significant differences between the UC and the CD group in age, weight, sex, or prior IV-IFX dosing and interval (Table 1). The age range was 5–17 years, including six patients younger than 12 years and five weighing less than 40 kg; all five patients weighing < 40 kg were also younger than 12 years. Among the 11 patients with CD, one patient (9%) had a perianal fistula and one patient (9%) had a clinically non-significant stricture of the terminal ileum prior to the switch; neither condition worsened during the observation period.

**Table 1:** Patient characteristics

	All (*n* = 20)	Ulcerative colitis (UC, *n* = 9)	Crohn’s disease (CD, *n* = 11)	*p*-value (UC vs. CD)
Age, years	14.5 (10.5–15.9)	13.6 (9.8–15.8)	15.4 (10.2–16.4)	0.31
Sex, female, n (%)	9 (45%)	6 (66.7%)	3 (27.3%)	0.18
Weight at switch, kg	53 (37.1–63.8)	54 (30.7–62.2)	53 (40.8–70.6)	0.79
Concomitant medication at switch, n (%)	8 (40%)	7 (78%)	1 (9%)	< 0.01
- azathioprine	4	3	1	
- mesalazine	6	6	–	
Time on IV-IFX before switch (weeks)	63 (17–136)	61 (13–132)	66 (27–156)	0.47
Dose of IV-IFX (mg/kg)	8 (6.25–9.5)	8 (7.5–9)	7 (6–10)	0.44
Interval of IV-IFX (every x weeks)	4 (4–6)	4 (4–6)	4 (4–6)	0.90
Observation time on SC-IFX, (weeks)	44 (26–60)	37 (27–61)	44 (20–60)	0.79
FC pre-switch (µg/g)	0 (0–154)	0 (0–119)	34 (0–270)	0.60
FC post-switch (µg/g)	0 (0–273)	0 (0–0)	88 (0–404)	0.21
IFX concentrations pre-switch, V0 (µg/mL)	11.8 (9–16.7)	13 (8.2–17.1)	10.6 (8.7–15.8)	0.36
IFX concentrations post-switch, V2 (µg/mL)	≥ 24 (range < 0.3–≥24) *n* = 12	≥ 24 (range < 0.3–≥24) *n* = 7	≥ 24 (range 15.5–≥24) *n* = 5	n.a.
shPCDAI pre-switch			0 (0–20)	
shPCDAI post-switch			0 (0–5)	
PUCAI pre-switch		0 (0–12.5)		
PUCAI post-switch		0 (0–10)		
Persistence (%)	16 (80%)	7 (77.8%)	9 (81.8%)	1.00

Values are n (%), n/n (%) or median (IQR Q1–Q3), unless otherwise specified. Statistical analysis with Fisher’s exact test and Mann-Whitney U test

*FC* Fecal calprotectin, *IFX* Infliximab, *IV-IFX* Intravenous infliximab, *n.a.* not applicable, *SC-IFX* Subcutaneous infliximab, *PUCAI* Pediatric ulcerative colitis activity index, *shPCDAI* Short pediatric Crohn’s disease activity index, *V0* Baseline visit, *V2* Second follow-up visit

Prior IV-IFX regimens were predominantly intensified: only 2 of 20 patients (10%) received the standard regimen of 5 mg/kg every 8 weeks, while the remaining 18 (90%) were on a higher dose and/or shortened interval (Table 1).

During IV-IFX therapy, eight patients were receiving concomitant therapy at a stable dose for at least 9 weeks prior to the switch: two received azathioprine only, four received mesalazine only, and two received both. All concomitant therapies were maintained unchanged at the time of the switch.

### SC-IFX treatment persistence

**Fig. 1:**
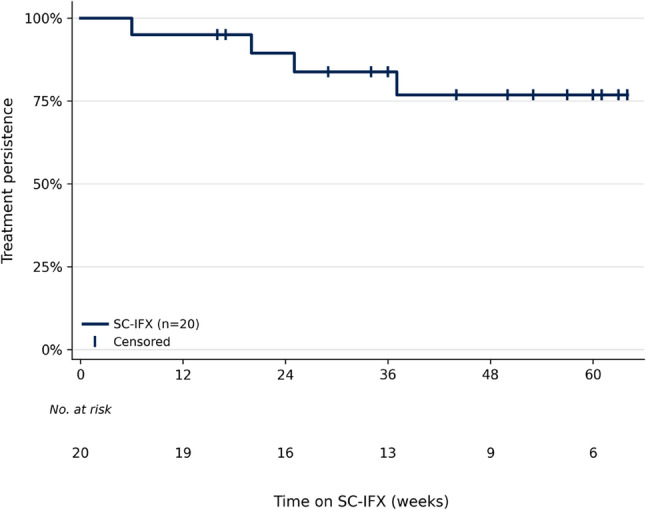
Kaplan-Meier estimate of SC-IFX treatment persistence. Censored observations are indicated by vertical marks. Numbers at risk are shown below the x-axis

Of the 20 patients who agreed to switch to SC-IFX, 16 (80%) remained on therapy after a median observation period of 44 weeks (IQR 26–60 weeks, see Table 1). Treatment persistence over time is illustrated in Fig. 1.

Persistence did not differ significantly between patients with UC (7/9, 77.8%) and CD (9/11, 81.8%) (Fisher’s exact test, *p* = 1.00). Four patients discontinued SC-IFX, and subsequent therapy was selected on a case-by-case basis at the discretion of the treating team. Two patients in clinical remission with respect to IBD but worsening of pre-existing paradoxical psoriasis discontinued after 6 and 20 weeks and were switched to adalimumab and methotrexate, respectively. One patient discontinued due to disease relapse and was switched to adalimumab. The fourth patient (IFX_07), who was initially in clinical remission but showed progressive loss of response with suspected non-adherence, was switched to vedolizumab.

Of the six patients younger than 12 years – five of whom also weighed < 40 kg – four (67%) remained on SC-IFX after a median observation period of 40 weeks (range 29–50 weeks). Among the five patients weighing < 40 kg, three (60%) remained on SC-IFX after a median observation period of 36 weeks (range 29–50 weeks). There was no significant weight difference between patients who discontinued SC-IFX (median 39.7 kg) and those who continued (median 53.5 kg; Z = − 1.371, *p* = 0.17, *r* = 0.30).

Five patients with mild clinical activity at switch (PUCAI 25–30 or shPCDAI 20) were included when practical circumstances warranted the switch (e.g., difficult IV access, long travel distances); four of these five (80%) achieved and maintained remission under SC-IFX treatment.

Three of four patients (75%) who received SC-IFX as a third dose following two doses of IV-IFX, maintained treatment over a median observation period of 60 weeks (range 16–64 weeks). One patient discontinued after 25 weeks due to progressive loss of response with suspected non-adherence; this patient was also receiving concomitant azathioprine. The three additional patients receiving concomitant azathioprine – all switched after a longer IV-IFX treatment period – maintained SC-IFX treatment over a median observation period of 60 weeks (range 17–64 weeks).

### Inflammatory markers pre and post switch

No significant differences were observed before and after the switch regarding inflammatory markers. For calprotectin and albumin, there was no significant difference (Z = − 0.31, exact *p* = 0.79, *r* = 0.10 and Z = − 0.87, exact *p* = 0.42, *r* = 0.24, respectively). For CRP, no significant difference was found either (Z = − 0.11, exact *p* = 1.00, *r* = 0.05). Effect sizes across all parameters were negligible to small (*r* = 0.05–0.24), suggesting no meaningful changes in these parameters after the switch.

### Positivity of anti-IFX antibodies

All three patients (one with UC and two with CD) with positive AIA before the switch became negative at V1 and remained negative while in clinical remission. None of these patients were receiving concomitant immunomodulatory therapy. No de novo AIA were detected in patients who had been negative prior to the switch.

### IFX concentrations – overall trends

At baseline (V0), the median IFX concentration was 11.8 µg/mL (IQR 9.0–16.7), with 2 of 20 patients (10%) at or above the ULOQ (≥ 24 µg/mL). At V2, 7 of 12 patients with available measurements (58%) had concentrations at or above the ULOQ; the corresponding V2 median was therefore ≥ 24 µg/mL (range < 0.3–≥24).

In one patient with UC (IFX_07), IFX concentrations continuously declined over time. This patient, who received SC-IFX as a third dose after two IV-IFX infusions, was initially in clinical remission and received SC-IFX for 25 weeks with concomitant azathioprine. As IFX concentrations at V0 were still adequate, non-adherence to the SC regimen at V1 and V2 cannot be excluded. Due to increasing disease activity and undetectable IFX concentrations, therapy was eventually switched to vedolizumab.

As an exploratory analysis, we compared pre-switch IV dose intensity (DI/BSA, mg/m²/week) — reflecting the dose required to maintain remission on IV-IFX — between patients who reached V2 IFX concentrations at or above the ULOQ (≥ 24 µg/mL, *n* = 7) and those who did not (*n* = 5). Patients who reached the ULOQ at V2 had a numerically lower median pre-switch DI/BSA (47.8 vs. 71.2 mg/m²/week), although this difference was not statistically significant (Mann-Whitney U = 7, *p* = 0.11).

### IFX concentrations – grouped by weight

**Fig. 2:**
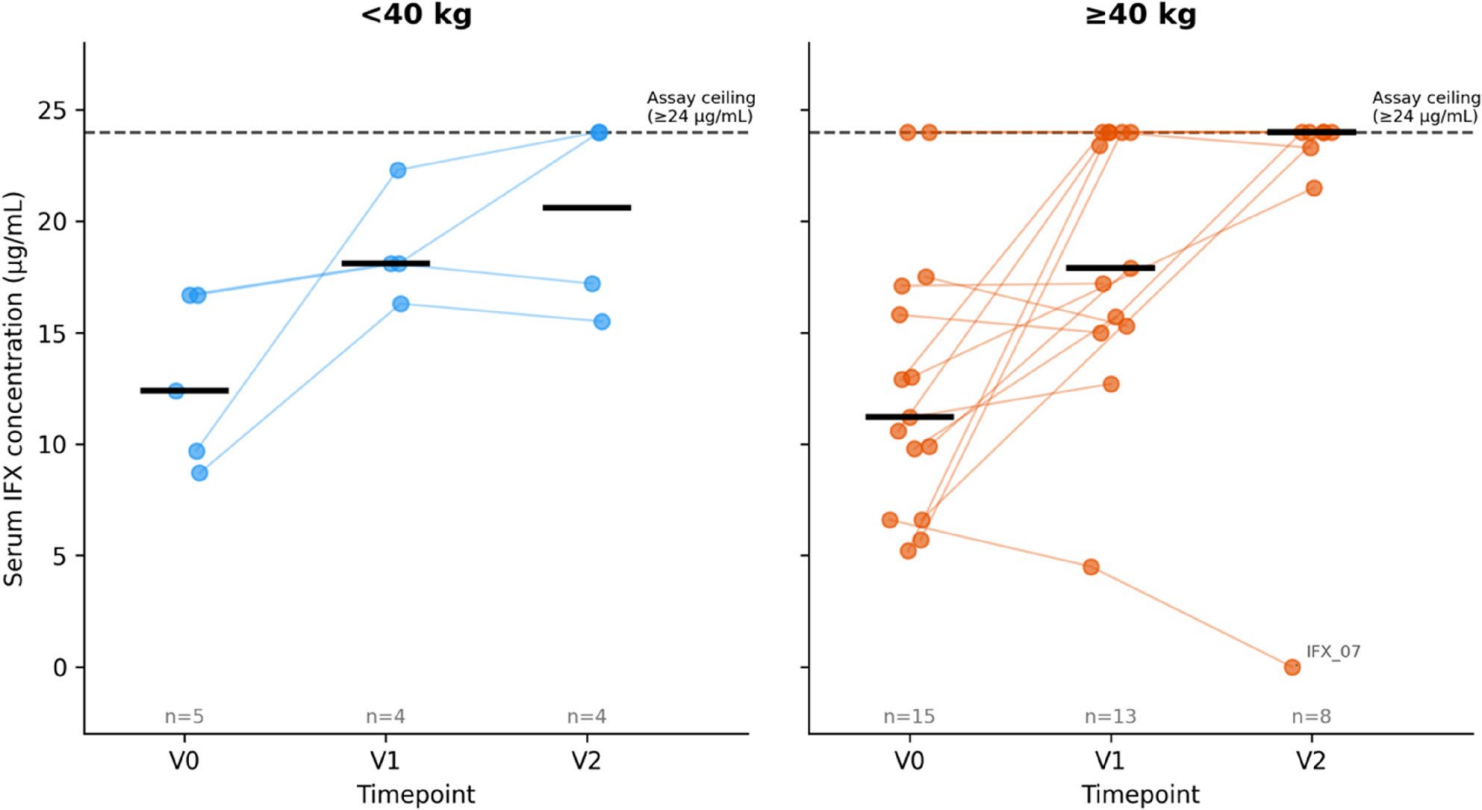
Serum IFX concentrations before and after switching to subcutaneous administration, grouped by weight. Each line represents an individual patient. Horizontal bars indicate the group median per timepoint. The dashed line indicates the upper limit of quantification of the assay (≥ 24 µg/mL); values at the ceiling are plotted at 24 µg/mL. Values below the lower limit of quantification (< 0.3 µg/mL) were plotted at 0 µg/mL for visualization only. IFX_07: patient with suspected non-adherence and undetectable IFX concentrations at V2 (see text)

Of the 20 patients switched, five had a weight < 40 kg. IFX concentrations were similar between the two weight groups (< 40 kg vs. ≥40 kg) across all visits (Fig. 2). Median IFX concentrations in the < 40 kg versus ≥ 40 kg group were 12.4 µg/mL versus 11.2 µg/mL at V0, 18.1 µg/mL versus 17.9 µg/mL at V1, and 20.6 µg/mL versus ≥ 24 µg/mL at V2. IFX concentrations grouped by age are shown in Supplementary Fig. 1.

### Tolerability

Two patients showed worsening of pre-existing paradoxical psoriasis; otherwise, no further adverse events were documented.

## Discussion

The rising global burden of pediatric IBD underscores the need for treatment strategies that are both effective and practical for children and adolescents. SC-IFX adds flexibility in treatment delivery tailored to individual patient needs, while intravenous administration remains available as an option. Direct evidence comparing the efficacy of SC-IFX and IV-IFX in pediatric patients remains limited, although available data suggest comparable effectiveness. Our findings indicate that SC-IFX may offer a viable alternative to intravenous therapy, addressing unmet needs particularly in younger and lower-weight patients, for whom data have so far been scarce.

We observed 80% treatment persistence with SC-IFX after a median observation period of 44 weeks, which is at the lower end of the 92–100% range reported in adult switching cohorts [6, 7] and below the 92% assumed a priori for sample-size calculation. However, it is broadly consistent with available pediatric real-world data: Boute et al. reported 78% persistence at 12 months in 66 children (median age 16.5 years) [10], while Duquerois et al. found 100% persistence at 6 months in 21 children (median age 17 years) [8]. Our cohort additionally included younger and lower-weight children, including patients as young as 5 years and weighing < 40 kg, as well as five patients with mild clinical activity at switch — factors that may explain the somewhat lower persistence relative to adult data.

Notably, this persistence rate was achieved across a broad range of prior IV-IFX dosing regimens, with the majority of patients not on the standard 5 mg/kg every 8 weeks. This suggests that switching to a fixed SC-IFX dose of 120 mg every two weeks may be feasible in this population, although exposure may be lower in patients with high prior dose intensity (explored below).

Paradoxical psoriasis is a well-recognized class effect of anti-TNF therapy in pediatric IBD patients [15]. Two patients in our cohort with pre-existing paradoxical psoriasis experienced worsening of skin symptoms within 20 weeks after switching to SC-IFX, ultimately leading to treatment discontinuation. Whether this worsening reflects the natural history of paradoxical psoriasis under continued IFX exposure or is specifically related to the subcutaneous route of administration remains unclear. To date, no adult or pediatric SC-IFX studies have specifically addressed outcomes in this subgroup.

The remaining two patients, who discontinued because of disease relapse or loss of response, were switched to adalimumab and vedolizumab, respectively. Notably, adalimumab is generally not the preferred option after non-pharmacokinetic infliximab failure, and its use here was driven by practical considerations (hospital-related anxiety, long travel distances).

In the subgroup of five patients weighing < 40 kg, treatment persistence was 60% over a median observation period of 36 weeks, and IFX concentrations were similar to those observed in heavier patients, suggesting adequate exposure despite the fixed-dose regimen. This is consistent with Boute et al., who reported 67% persistence at 12 months in six patients weighing < 40 kg with no significant difference in IFX concentrations [10]. Such exposure is clinically relevant, since higher SC-IFX concentrations (> 20 µg/mL) have been associated with deep remission in adult IBD [16], and higher post-induction IFX concentrations are linked to favorable clinical outcomes in pediatric Crohn’s disease [17].

Regarding the use of SC-IFX as a third dose following induction, Eldredge et al. reported that six pediatric patients received SC-IFX as their third dose, in accordance with adult treatment protocols, although persistence outcomes for this subgroup were not specified. In our cohort, four patients received SC-IFX as a third dose, and 3/4 patients maintained treatment persistence over follow-up. This observation aligns with adult data [7], suggesting that switching to SC-IFX could be considered early during maintenance therapy in selected pediatric patients.

Consistent with the established pharmacokinetic profile of SC-IFX, serum IFX concentrations increased after the switch from IV-IFX, with 7 of 12 patients with available measurements (58%) reaching the ULOQ (≥ 24 µg/mL) by the V2 assessment (median 16 weeks post-switch). This trajectory mirrors observations in adult cohorts, in which median trough concentrations rose after the switch — for example from approximately 8.9 µg/mL to 16 µg/mL [7] — and is consistent with pediatric data: Boute et al. reported an increase in median concentrations from 6.6 to 13.9 µg/mL (*p* = 0.02) [10], Eldredge et al. a rise from 7.8 to 19.6 µg/mL (*p* < 0.001) [11], and Duquerois et al. an increase from 11.3 µg/mL at switch to approximately 17–20 µg/mL during SC-IFX therapy [8]. Direct quantitative comparison across cohorts is limited by differing assay ceilings and right-censoring. Nevertheless, the higher ULOQ in the present study (24 µg/mL), compared with 12–20 µg/mL in Boute et al., allowed better characterization of the upper post-switch concentration range before ceiling effects occurred; assay ceilings were not reported by Eldredge et al. or Duquerois et al. [8, 10, 11].

Patients who had required more intensive IV-IFX dosing before the switch showed numerically lower SC-IFX exposure, although this exploratory finding was not statistically significant. This may reflect persistent pharmacokinetic characteristics, such as higher drug clearance, and is consistent with simulations by Weersink et al. predicting reduced SC-IFX exposure after switching from high-frequency IV dosing [18]. Together with adult data suggesting higher relapse rates after switching from intensified IV regimens [19], these findings support closer therapeutic drug monitoring in this subgroup.

A noteworthy finding of our study was the favorable immunogenicity profile observed after switching to SC-IFX. All three patients with detectable AIA prior to the switch became antibody-negative during SC-IFX therapy while remaining in clinical remission, and no de novo AIA formation was observed. Similarly, Boute et al. reported resolution of pre-existing AIA in three of four patients under SC-IFX treatment, although two patients developed de novo AIA while remaining in clinical remission [10]. These findings are compatible with the hypothesis that the more stable, continuous drug exposure provided by subcutaneous administration reduces detectable immunogenicity. In adult studies, comparable rates of AIA have been reported for IV-IFX and SC-IFX, indicating no increased immunogenic risk with the subcutaneous formulation [12].

A recent meta-analysis suggests that concomitant treatment with thiopurines or methotrexate may reduce immunogenicity during SC-IFX therapy, while clinical responses appear comparable [20]. In our cohort, only 4/20 patients received azathioprine, which was continued unchanged across the switch; this was too few for meaningful subgroup analysis. However, the predominance of monotherapy makes concomitant immunomodulation an unlikely primary driver of our findings. AIA results should nonetheless be interpreted in light of substantial inter-assay variability [21]: drug-sensitive assays detect antibodies only at low IFX concentrations, whereas the drug-tolerant CLIA assay used in this study is less affected by circulating drug, limiting cross-study comparability.

Several limitations should be acknowledged. The small sample size and retrospective design inherently constrain causal interpretation. However, in pediatric inflammatory bowel disease, access to biologic therapies frequently lags adult indications due to delayed approval pathways [22], underscoring the relevance of real-world observational data to inform clinical practice until prospective pediatric studies become available. As patients in clinical remission were prioritized for switching, the cohort may reflect a more favorable disease activity profile than the general pediatric IBD population, limiting generalizability. Another limitation is that the timing of blood sampling relative to the last SC-IFX injection was not recorded, resulting in the assessment of random IFX serum concentrations. In addition, IFX serum concentrations could only be quantified up to 24 µg/mL due to assay limitations, potentially limiting the assessment of higher drug exposure concentrations. Future studies employing assays with a higher ULOQ would allow more precise characterization of SC-IFX steady-state exposure.

Furthermore, the retrospective design precluded systematic prospective assessment of adverse events, and mild or transient reactions may have gone unreported. Finally, patient and caregiver satisfaction with the switch to SC-IFX was not assessed using standardized quantitative measures; however, informal feedback was consistently positive and aligns with previously reported high satisfaction rates and reductions in missed school and work days in pediatric cohorts [10, 11].

## Conclusion

In this real-world pediatric cohort, SC-IFX demonstrated good treatment persistence, adequate drug exposure, and a favorable immunogenicity profile, extending previous findings to younger and lower-weight children. Our data support the feasibility of SC-IFX as a maintenance option in selected pediatric patients, including early use after induction. While a fixed-dose regimen appears acceptable in routine practice, further prospective studies are needed to define pediatric-specific pharmacokinetic targets and to develop individualized treatment strategies.

## Supplementary Information

Supplementary Material 1: Supplementary Fig. 1. Serum IFX concentrations before (V0) and after switching to subcutaneous administration (V1, V2), grouped by age. Each line represents an individual patient; horizontal bars indicate the group median per timepoint. The dashed line indicates the upper limit of quantification of the assay (≥ 24 µg/mL); values at the ceiling are plotted at 24 µg/mL. Values below the lower limit of quantification (< 0.3 µg/mL) were plotted at 0 µg/mL for visualization only. IFX_07: patient with suspected non-adherence and undetectable IFX concentrations at V2.

## Data Availability

Anonymized data will be available from the corresponding author on reasonable request.

## Abbreviations

AIA: Anti-infliximab antibodies
BSA: Body surface area
CD: Crohn’s disease
CI: Confidence interval
CLIA: Chemiluminescence immunoassay
CRP: C-reactive protein
DI: Dose intensity
FC: Fecal calprotectin
IBD: Inflammatory bowel disease
IFX: Infliximab
IQR: Interquartile range
IV-IFX: Intravenous infliximab
LLOQ: Lower limit of quantification
PUCAI: Pediatric ulcerative colitis activity index
SC-IFX: Subcutaneous infliximab
SD: Standard deviation
shPCDAI: Short pediatric Crohn’s disease activity index
TNF: Tumor necrosis factor
UC: Ulcerative colitis
ULOQ: Upper limit of quantification
