# Supplementary figures and images for: Subcutaneous infliximab as a maintenance option in pediatric IBD: a real-world cohort including younger and lower-weight children
Source: Mol Cell Pediatr. 2026 Jul 17;13:38. doi: 10.1186/s40348-026-00251-2 (PMC13376280; doi:10.1186/s40348-026-00251-2)

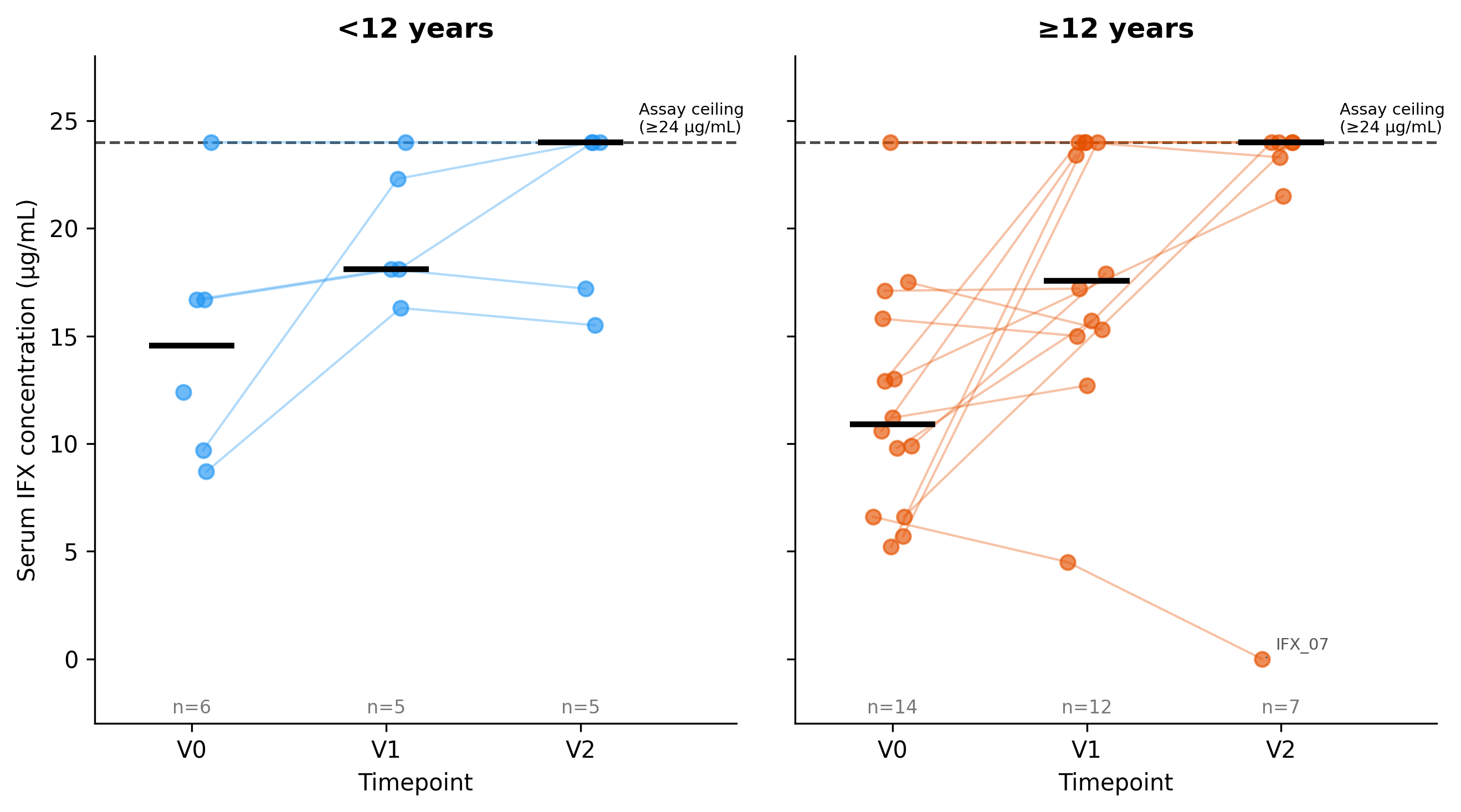

Supplement: Supplementary file 1 — Supplementary Material 1: Supplementary Fig. 1. Serum IFX concentrations before (V0) and after switching to subcutaneous administration (V1, V2), grouped by age. Each line represents an individual patient; horizontal bars indicate the group median per timepoint. The dashed line indicates the upper limit of quantification of the assay (≥ 24 µg/mL); values at the ceiling are plotted at 24 µg/mL. Values below the lower limit of quantification (< 0.3 µg/mL) were plotted at 0 µg/mL for visualization only. IFX_07: patient with suspected non-adherence and undetectable IFX concentrations at V2. [file 40348_2026_251_MOESM1_ESM.png]
